# The Burden of COPD Morbidity Attributable to the Interaction between Ambient Air Pollution and Temperature in Chengdu, China

**DOI:** 10.3390/ijerph15030492

**Published:** 2018-03-11

**Authors:** Hang Qiu, Kun Tan, Feiyu Long, Liya Wang, Haiyan Yu, Ren Deng, Hu Long, Yanlong Zhang, Jingping Pan

**Affiliations:** 1 Health Big Data Research Institute, Big Data Research Center, University of Electronic Science and Technology of China, Chengdu 611731, China; qiuhang@uestc.edu.cn (H.Q.); hyyu@uestc.edu.cn (H.Y.); 2 Health and Family Planning Information Center of Sichuan Province, Chengdu 610041, China; tankun@schnic.cn (K.T.); longhu@schnic.cn (H.L.); panjingping@schnic.cn (J.P.); 3 Sichuan Health Information Association, Chengdu 610041, China; 4 School of Electronic and Information Engineering, Beijing Jiaotong University, Beijing 100044, China; 14211012@bjtu.edu.cn; 5 School of Economics and Management, Chongqing University of Posts and Telecommunications, Chongqing 400065, China; 6 Department of Statistics, The Pennsylvania State University, University Park, PA 16802-2111, USA; 7 Chengdu Shulianyikang Technology Co., Ltd., Chengdu 610041, China; zhangyanlong@shulianyikang.com

**Keywords:** air pollution, temperature, COPD, interaction, hospital admissions

## Abstract

Evidence on the burden of chronic obstructive pulmonary disease (COPD) morbidity attributable to the interaction between ambient air pollution and temperature has been limited. This study aimed to examine the modification effect of temperature on the association of ambient air pollutants (including particulate matter (PM) with aerodynamic diameter <10 μm (PM_10_) and <2.5 μm (PM_2.5_), nitrogen dioxide (NO_2_), sulfur dioxide (SO_2_), carbon monoxide (CO) and ozone (O_3_)) with risk of hospital admissions (HAs) for COPD, as well as the associated morbidity burden in urban areas of Chengdu, China, from 2015 to 2016. Based on the generalized additive model (GAM) with quasi-Poisson link, bivariate response surface model and stratification parametric model were developed to investigate the potential interactions between ambient air pollution and temperature on COPD HAs. We found consistent interactions between ambient air pollutants (PM_2.5_, PM_10_ and SO_2_) and low temperature on COPD HAs, demonstrated by the stronger associations between ambient air pollutants and COPD HAs at low temperatures than at moderate temperatures. Subgroup analyses showed that the elderly (≥80 years) and males were more vulnerable to this interaction. The joint effect of PM and low temperature had the greatest impact on COPD morbidity burden. Using WHO air quality guidelines as reference concentration, about 17.30% (95% CI: 12.39%, 22.19%) and 14.72% (95% CI: 10.38%, 19.06%) of COPD HAs were attributable to PM_2.5_ and PM_10_ exposures on low temperature days, respectively. Our findings suggested that low temperature significantly enhanced the effects of PM and SO_2_ on COPD HAs in urban Chengdu, resulting in increased morbidity burden. This evidence has important implications for developing interventions to reduce the risk effect of COPD morbidity.

## 1. Introduction

Chronic obstructive pulmonary disease (COPD) is well-known as one of the many non-communicable diseases with high morbidity and mortality [1,2]. The World Health Organization (WHO) has announced that more than 3 million deaths were attributable to COPD in 2015, which accounted for 5% of all global deaths in that year [3]. In China, the number of COPD cases increased to 54.8 million in 2013. In the same year, there were 910,809 deaths from COPD in China, representing 31.1% of the total deaths from COPD in the world [4]. 

The effects of ambient air pollution on patients suffering from COPD have been widely reported [5]. Consistent findings have indicated that outdoor air pollutants played important roles in the occurrence and exacerbation of COPD [6,7], resulting in increased rates of mortality [8], outpatient visits [9], emergency department visits [10] and hospital admissions (HAs) [11]. However, previous studies investigating health effects of ambient air pollution have traditionally controlled for temperature as a confounder. It remains uncertain whether pollutants’ effects are modified by temperature.

Recently, an increasing number of studies have begun to explore the potential interaction between temperature and air pollutants or effect modification [12,13,14,15]. Nevertheless, most of these studies focused on mortality. Very few studies have examined the joint effect between ambient air pollution and temperature on nonfatal health outcomes such as HAs, and these few studies produced inconsistent results. For instance, a study in Taiwan [16] found an increased risk of emergency department visits and HAs for COPD associated with ambient air pollution on higher temperature days, while studies in Hong Kong [17,18] and the United States [19] reported increased effect on cold days. 

More importantly, the burden of COPD morbidity associated with the interaction between ambient air pollutants and temperature has rarely been reported, particularly in a developing country such as China. Given interaction between ambient air pollution and temperature on COPD HAs to evaluate attributable burden for COPD morbidity is better than temperature as a confounder [15]. Compared with ratio measures, such as relative risk (RR) and odds ratio (OR), the disease burden may provide more relevant information for policy-makers, help them comprehensively assess the synergistic effects of outdoor air pollution and temperature, and design intervention strategies. 

In the present study, we aimed to examine the modification effect of temperature on the relationship between major ambient air pollutants (including particulate matter (PM) with aerodynamic diameter <10 µm (PM_10_) and <2.5 µm (PM_2.5_), nitrogen dioxide (NO_2_), sulfur dioxide (SO_2_), carbon monoxide (CO) and ozone (O_3_)) and COPD HAs among residents in urban areas of Chengdu between 2015 and 2016; we further estimated the morbidity burden using attributable fraction (AF) and attributable number (AN). 

## 2. Materials and Methods

### 2.1. Study Area

Located in the southwest of China, Chengdu is the largest and most densely populated city in the Sichuan Basin (latitude 30°05′–31°26′ N and longitude 102°54′–104°53′ E). Our study area involved five urban districts of Chengdu City: Jinjiang, Qingyang, Jinniu, Wuhou and Chenghua, covering an area of 465 square kilometers. There were about 3.72 million permanent residents within the area at the end of 2015. The city has subtropical climate characteristics, with an annual average temperature of 16.2 °C. In addition, there are more clouds and mist, and less sunlight, with heavy humidity and mild wind speed.

### 2.2. Data Collection

HAs data for COPD from 1 January 2015 to 31 December 2016 were obtained from the database of Health and Family Planning Information Center of Sichuan Province (HFPI-SC). HFPI-SC collects electronic hospitalization summary reports (HSRs) from the Electronic Medical Record System of all the tertiary hospitals and secondary hospitals in Sichuan Province [20]. These records contain the date of birth, age, gender, residential address, the date of admission and discharge, primary discharge diagnosis, and up to 15 secondary discharge diagnoses. The criteria for data extraction in our study include: (1) a primary diagnosis of COPD (International Classification of Diseases, 10th Revision codes: J41–J44); (2) residential addresses of the patients are in urban districts of Chengdu; (3) HAs data from tertiary hospitals and secondary hospitals.

Ambient air quality data were derived from the web platform of the China National Environmental Monitoring Center (http://www.cnemc.cn/) managed by the Ministry of Environmental Protection of the People’s Republic of China. Data of hourly air pollution concentrations of PM_2.5_, PM_10_, SO_2_, NO_2_, CO and O_3_ from 6 air quality monitoring stations interspersed in five urban districts of Chengdu city were obtained. All areas of the five urban districts were located within 40 km radius of the monitoring stations. It has been suggested that the monitoring data could be used as a proxy for personal exposure [21,22,23]. The daily concentrations of air pollutants were calculated as the 24-h mean concentration, except for O_3_, which was calculated as the maximum 8-h moving average under the China National Quality Control [24,25]. When the data were missing for a particular monitoring station on a given day, the values from the remaining stations were used to compute the 24-h average or maximum 8-h moving average. The linear interpolation, a common method for imputing missing time-series data in environmental research [26,27], was used for each monitoring station before statistical analyses when the data were missing (missing rate: 4.79%, 35/731) for all the 6 monitoring stations. It has been reported that the linear interpolation has acceptable performance and reliability especially when the percentage of missing values are low (e.g., <5%) [28].

Meteorological data, including daily average temperature and relative humidity, were collected from the Chengdu Meteorological Monitoring Database (http://data.cma.cn/).

### 2.3. Statistical Methods

#### 2.3.1. Core Model Development

The time-series approach was used to assess the effects of ambient air pollution on COPD HAs. Because it is generally assumed that daily COPD HAs followed the over-dispersed Poisson distribution, we firstly built the core model based on generalized additive model (GAM) with quasi-Poisson link without air pollution and temperature data [29]. The potential confounding effects of daily relative humidity, long-term trend and seasonality of COPD HAs were controlled by using a penalized spline approach based on generalized cross-validation (GCV). The initial value of degree of freedom (df) for each smoothing variable was automatic selected based on effect degree of freedom (edf), which means that if the relative risk did not change beyond the present value, then we regarded it as the “best” degree of freedom [30,31,32]. Public holiday and day of week were adjusted through the dummy variables in the core model:

(1) $$Log[E(Y_{t})]=\alpha +s(Time_{t})+s(RH_{t})+DOW_{t}+Holiday_{t}$$

where $E(Y_{t})$ presents daily COPD HAs on day *t*; *α* is the intercept; $s(Time_{t})$ and $s(RH_{t})$ denote the smoothing function for nonlinear variables, which represent the calendar time and relative humidity on day *t*, respectively; $DOW_{t}$ is day of week; $Holiday_{t}$ is a public holiday.

#### 2.3.2. Analysis of Individual Effects

Before examining the modification effect of temperature, analysis of individual effects of PM_2.5_, PM_10_, SO_2_, NO_2_, CO and O_3_ were developed through adding the daily concentrations into the core model and temperature as a confounder to explore the association between ambient air pollutants and COPD HAs. The lag effects of both single-day (from lag 0 to lag 6) and multiple-day moving average (from lag 01 to lag 06) were also taken into consideration to evaluate which of the lag day had the strongest association with COPD HAs. The individual effect model is shown as follows:

(2) $$Log[E(Y_{t})]=\alpha +\beta_{1}*AP_{t-i}+\beta_{2}*Tem_{t}+COVs$$

where $AP_{t-i}$ stands as the concentration of ambient air pollutants (PM_2.5_, PM_10_, SO_2_, NO_2_, CO and O_3_) on day *t* − *i*; *Tem_t_* is mean temperature on day *t*; *COVs* denotes all the confounders in the core model (1). The excess relative risk was expressed as the percentage change (PC) and 95% confidence intervals (95% CI) in daily COPD HAs with 10 μg/m^3^ increase in daily ambient air pollutants (except for CO per 0.1 mg/m^3^ ) based on the relative coefficient (*β*_1_). The lag day with statistically significant (*p* < 0.05) and the strongest PC would be taken into account for further interaction analysis and calculating COPD morbidity burden.

#### 2.3.3. Analysis of Interaction Effects

Two approaches were applied to investigate the possible effect modification by temperature, while accounting for potential confounders in core model (1). 

Firstly, we fitted a bivariate response surface model to visually estimate the potential interactive patterns of ambient air pollutants and temperature on COPD HAs. The concentrations of ambient air pollutants and mean temperature with one smoothing spline was added to the core model (1) to develop the bivariate response surface model:

(3) $$Log[E(Y_{t})]=\alpha +te(AP_{t-i},Tem_{t})+COVs$$

where $te(AP_{t-i},Tem_{t})$ represents the joint effect of ambient air pollutants and temperature at the lag days (*i*) selected in individual effect model; *COVs* stands as the same confounders in core model (1).

Secondly, a temperature-stratified parametric model was developed to examine whether the effects of ambient air pollutants on COPD HAs were heterogeneous across different temperature levels:

(4) $$Log[E(Y_{t})]=\alpha +\beta *(AP_{t-i}:Tem_{t}level)+COVs$$

where *β* is the relative coefficient across different temperature levels, which is used to calculate PC (%) and 95% CI for each 10 μg/m^3^ increase in the concentration of air pollutants (except for CO per 0.1 mg/m^3^ ) across different temperature levels, and further calculate AN and AF across different temperature levels; $Tem_{t}level$ is defined as mean temperature ($Tem_{t}$) categorized into three levels (low, moderate and high) using the different cut-off points. The selection of the cut-off points is arbitrary. In this model, we test cut-off points of temperature ranging from the 5th and 95th to the 25th and 75th percentiles by increments of five percentiles and selected the 20th (9.3 °C) and 80th (24 °C) percentiles as they produced models with the lowest GCV score [8] (Supplementary Materials Table S1).

#### 2.3.4. Estimating COPD Morbidity Burden 

To estimate the COPD morbidity burden attributable to the interaction between ambient air pollution and temperature, the WHO air quality guidelines [33,34], China grade II standard for air quality [25,35] and 50% China grade II standard were considered as the references, respectively. The AF and AN were calculated by effect estimates of ambient air pollutants concentrations for each temperature level according to the stratification parametric model (4) [36], using the following equations:

(5) $$AN_{t}=n_{t}*B\_AF_{t}$$

(6) $$B\_AF_{t}=1-\exp(-\beta *\Delta AP_{t-i})$$

where $AN_{t}$ is the number of COPD HAs attributed to PM on day *t*; $n_{t}$ is the reported number of COPD HAs on day *t*; $B\_AF_{t}$ is the attributable fraction due to moving average effects on day *t*; *β* is the effect estimates across different temperature levels in model (4); $\Delta AP_{t-i}$ is the concentration difference between the observed concentrations and reference concentrations on day *t* − *i*. The overall AF was assessed by dividing the sum of AN in each temperature level by the total number of COPD HAs. 95% CI of *β* were used to calculate the 95% CI of AF and AN with the above equation [12].

#### 2.3.5. Sensitivity Analysis

As temperature is associated with health outcomes and the effect may last for several days, the potential effects of temperature on health outcomes in previous studies were estimated with current day, moving average of the current day and previous three days [12,24,37]. Different cut-off points (5th and 95th; 10th and 90th; 15th and 85th; 20th and 80th; 25th and 75th percentiles) of temperature on moving average of the current day and previous three days were used to test the modification effect of temperature on association between ambient air pollution and COPD HAs. To identify subgroups vulnerability to ambient air pollution exposure in each temperature level, we stratified HAs by age (<60 years, 60–70 years, 70–80 years and ≥80 years) and gender (male and female), and repeated the analyses in each subgroup. The Z-test was applied to test the statistical significance of the differences by age and gender [15]. To further test the robustness of the effect estimates, co-pollutant models were performed based on model (4) and adjusted for another air pollutant, which was significant in the individual effect model [24]. Considering that the air pollutants were correlated (Supplementary Materials Table S2) and might be interactive with each other, only those with Spearman correlation coefficients below 0.8 were admitted to the co-pollutant model [18,38]. All statistical analyses were conducted in R software (version 3.4.0, R Development Core Team, Vienna, Austria).

## 3. Results

Table 1 shows the descriptive results. During the study period, a total of 54,966 COPD HAs (male:female ratio = 1.8:1) with residential address in the urban areas of Chengdu were recorded from 124 hospitals (including tertiary hospitals and secondary hospitals) in Chengdu, 59 of them located in the urban areas. Daily admission counts for the elderly (≥60 years) accounted for 91.8%. Over the 731 days of the study, there were approximately 75 COPD HAs (range: 15 to 194) per day. The mean daily concentrations of ambient air pollutants were 57.29 μg/m^3^ for PM_2.5_, 94.73 μg/m^3^ for PM_10_, 13.80 μg/m^3^ for SO_2_, 50.49 μg/m^3^ for NO_2_, 1.07 mg/m^3^ for CO and 96.73 μg/m^3^ for O_3_. Of the total days, 87.81%, 83.40% and 64.65% of the days exceeded the WHO air quality guidelines for PM_2.5_, PM_10_ and SO_2_ (25 μg/m^3^, 50 μg/m^3^ and 20 μg/m^3^, respectively) [33], and 23.4%, 16.0%, 5.3% and 15.5% of the days exceeded the China grade II standard for PM_2.5_, PM_10_, NO_2_ and O_3_ (75 μg/m^3^, 150 μg/m^3^, 80 μg/m^3^ and 160 μg/m^3^, respectively) [25]. The mean daily concentrations of SO_2_ and CO were well below the China grade II standard (150 μg/m^3^ and 4 mg/m^3^ , respectively) [25].

Figure S1 shows the individual effects of air pollutants. PM_2.5_, PM_10_, SO_2_, NO_2_ and CO were positively associated with COPD HAs, with the strongest effects at lag 06, lag 05, lag 05, lag 05 and lag 05, respectively. These lag days were chosen for further interaction analysis and evaluation of COPD morbidity burden. The associations between O_3_ and COPD HAs were insignificant at both single-day (from lag 0 to lag 6) and multiple-day moving average (from lag 01 to lag 06). 

**Figure 1:**
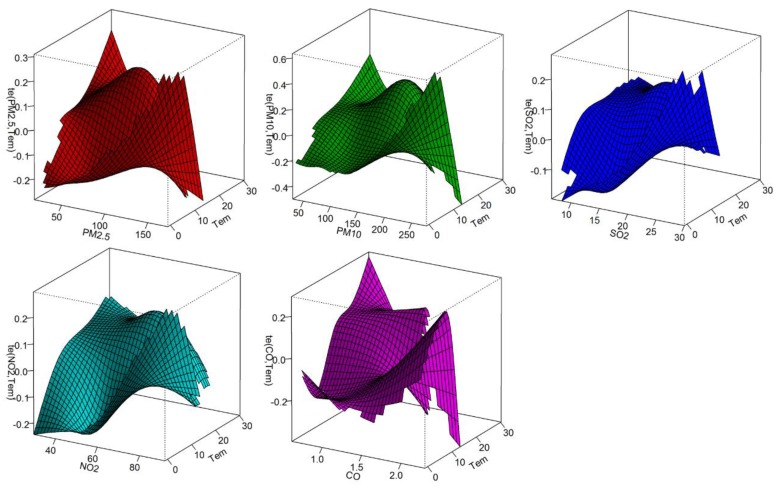
Interactive effects between air pollutants and temperature on COPD HAs.

Figure 1 shows the potential interactive effects of ambient air pollutants and temperature on COPD HAs, using joint response surfaces. When ambient air pollutants are at high levels and temperature at low levels, the risk of COPD HAs peaked. 

Table 2 shows percentage change of daily COPD HAs per 10 μg/m^3^ increase (CO per 0.1 mg/m^3^ ) in ambient air pollutants concentrations across three levels of temperature using the 20th (9.3 °C) and 80th (24 °C) percentiles as the cut-off points. There were interactive effects between ambient air pollutants (PM_2.5_, PM_10_, SO_2_ and NO_2_) and low temperature on COPD HAs, as demonstrated by the consistently stronger effects of ambient air pollutants in the low temperature level than those in the moderate temperature level (*p* < 0.05). In co-pollutant models, the temperature-stratified effects of PM_2.5_, PM_10_ and SO_2_ were attenuated by co-pollutant, and the effect of NO_2_ became insignificant. For example, on low temperature (≤20th percentile) days, a 10 μg/m^3^ increment in PM_2.5_, PM_10_, SO_2_ and NO_2_ corresponded to a 2.90% (95% CI: 2.07%, 3.73%), 1.73% (95% CI: 1.22%, 2.25%), 17.04% (95% CI: 10.77%, 23.66%) and 5.59% (95% CI: 3.65%, 7.56%) increase in COPD HAs, respectively, which was significantly higher than that on moderator temperature days. After adjustment for co-pollutant (such as CO), the estimates decreased to 2.25% (95% CI: 1.37%, 3.13%), 1.31% (95% CI: 0.75%, 1.86%), 11.83% (95% CI: 5.59%, 18.44%) and 3.48% (95% CI: 1.37%, 5.64%), respectively, and the difference compared with the estimates on moderate days still significant for PM_2.5_, PM_10_ and SO_2_. 

**Figure 2:**
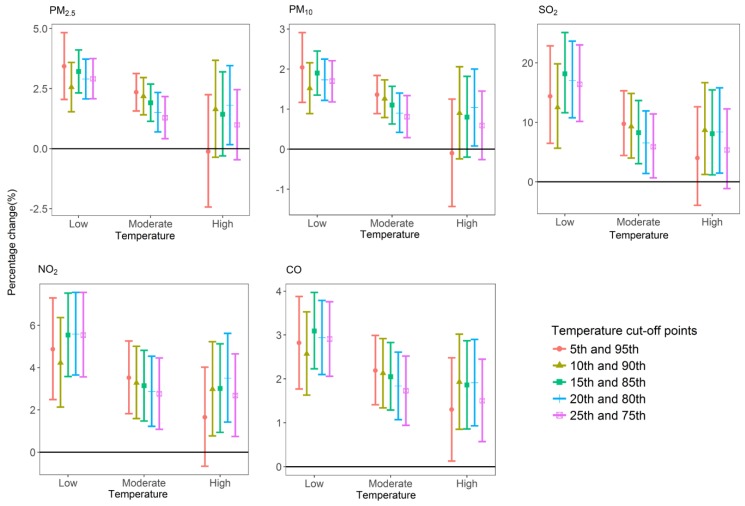
Associations between daily air pollutant concentrations and COPD HAs stratified by varying percentiles of temperature cut-off points.

Figure 2 and Figure S2 show different temperature (current day, moving average of the current day and previous three days) stratification on interaction estimates, using different cut-off points (5th and 95th; 10th and 90th; 15th and 85th; 20th and 80th; 25th and 75th percentiles). The results were consistent when using moving average of the current day and previous three days’ temperature, which suggested that the modification effects between air pollutants and low temperature on COPD HAs were robust. The use of alternative cut-off points for temperature levels yielded similar trends, but the magnitude of the interaction differed. 

**Figure 3:**
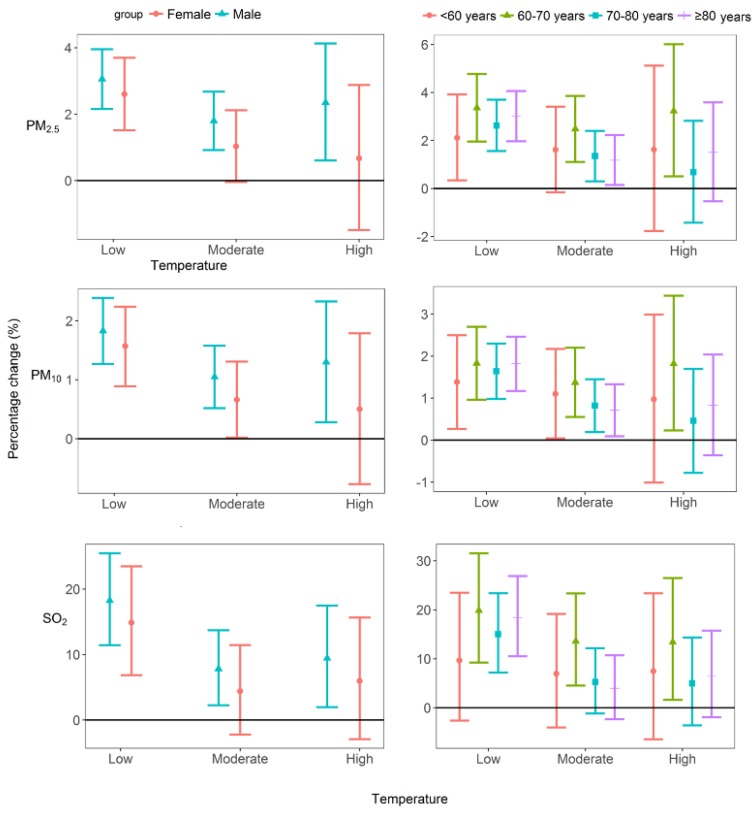
Associations between PM_2.5_/PM_10_/SO_2_ concentrations and COPD HAs in low, moderate and high temperature level by age and gender. The percentage change of daily COPD HAs associated with a 10 μg/m^3^ increase in PM_2.5_, PM_10_ and SO_2_ concentrations.

Figure 3 and Figure S3 compare temperature modification effects in different age and gender groups using 20th and 80th percentiles as the cut-off points. The consistently stronger interactive effects between ambient air pollutants (PM_2.5_, PM_10_ and SO_2_) and low temperature on COPD HAs in the elderly population aged above 80 years were observed, with an increase of 10 µg/m^3^ in PM_2.5_, PM_10_ and SO_2_ corresponded to 3.01% (95% CI: 1.97%, 4.06%), 1.82% (95% CI: 1.17%, 2.46%) and 18.42% (95% CI: 10.52%, 26.89%) increases in COPD HAs. Within the elderly subgroup, the effects of ambient air pollutants in the low temperature level were consistently stronger than that in the moderate temperature level. For other age groups, such differences were less prominent. In terms of the differences in gender groups, the interactive effects between ambient air pollutants and low temperature on COPD HAs were stronger in males than in females, but these differences were insignificant (*p* > 0.05). Within the male and female subgroups, the effects of ambient air pollutants on low temperature days were consistently stronger than that on moderate temperature days (*p* < 0.05).

Table 3 shows the estimated AF and AN of COPD HAs due to PM_2.5_, PM_10_ and SO_2_ exposures, which demonstrated modification effects across different temperature levels in the above steps. The highest AF consistently occurred at low temperature levels. For example, when using WHO air quality guidelines as the reference concentration, about 17.30% (95% CI: 12.39%, 22.19%), 14.72% (95% CI: 10.38%,19.06%) and 1.14% (95% CI: 0.74%, 1.54%) of COPD HAs on low temperature days could be attributed to exceeding daily PM_2.5_, PM_10_ and SO_2_ concentrations, respectively, but only 4.77% (95% CI: 2.21%, 7.33%), 4.16% (95% CI: 1.92%, 6.41%) and 0.08% (95% CI: 0.02%, 0.14%) at moderate temperature levels. Comparing to China grade II standard, about 5.89% (95% CI: 4.22%, 7.57%) and 3.35% (95% CI: 2.36%, 4.33%) of COPD HAs at low temperature levels may result from exposure to PM_2.5_ and PM_10_, respectively, which was approximately 9.2 and 12.0 times higher than that at moderate temperature levels, respectively. As daily concentration of SO_2_ was well below the China grade II standard and 50% China grade II standard, AF and AN attributable to SO_2_ exposure across different temperature levels was zero.

## 4. Discussion

Although the associations between ambient air pollutants and COPD morbidity have been well documented worldwide, few studies have investigated the interaction between ambient air pollution and temperature on COPD morbidity, and little is known regarding the morbidity burden attributable to this interaction. This study used time-series analyses to investigate the joint effect of ambient air pollution and temperature on COPD HAs in urban areas of Chengdu, China, and found that low temperature significantly enhanced the effects of PM_2.5_, PM_10_ and SO_2_ on COPD HAs, and the joint effect of PM_2.5_/PM_10_ and low temperature had the greatest impact on the COPD morbidity burden. To the best of our knowledge, this is the first study to estimate the burden of COPD morbidity due to this interaction in southwestern China.

It is now accepted that PM is positively associated with COPD HAs, and PM_2.5_ is more harmful to health outcome than PM_10_ because the former can be inhaled more deeply into the small airways of the lung [5,39,40]. However, evidence on the interaction between PM and temperature on COPD HAs, especially in basin areas, was limited. Consistent with previous studies in Hong Kong [17,18] and the United States [19], we found a significant and positive interactive effect between PM_2.5_/PM_10_ and low temperature on COPD HAs, with a considerable increase of 2.90% (95% CI: 2.07%, 3.73%) and 1.73% (95% CI: 1.22%, 2.25%) per 10 μg/m^3^ increase of PM_2.5_ and PM_10_ on low temperature days (≤20th percentile), respectively, which was statistically stronger than that on moderate temperature days (20th–80th percentile). Ding et al. [16] demonstrated a significantly and greater effect for PM_2.5_ (OR = 1.037, 95% CI: 1.001, 1.074) coexisted with O_3_ exposure in the days with higher temperature (>27.9 °C) on COPD-related emergency department visits in Taiwan. Located in the bottom of the Sichuan Basin and surrounded with huge mountains, Chengdu has typical basin climate characteristics of high humidity, static wind frequency and atmospheric stability subject to neutral weather in winter, which obstruct the air pollutants’ transport and diffusion, form local circulation and then result in continuous heavy pollution weather, especially heavy PM accumulation [41,42]. These special topographic and climatic conditions, as well as differences in health endpoints and modeling strategies, may contribute to this heterogeneity of findings, compared with previous studies.

With regards to the gaseous pollutants, only SO_2_ presented interactive effect with low temperature, with a 10 μg/m^3^ increment associated with an 11.22% (95% CI: 4.70%, 18.14%) increase in COPD HAs after adjustment for PM_2.5_. This result was much higher than the study in Hong Kong, which demonstrated that a 10 μg/m^3^ increment of SO_2_ was associated with 1.99% (95% CI: 0.90%, 3.09%) increase in emergency COPD HAs on the cool and dry days [18]. Differences among the effect estimates of SO_2_ may be related with variations of the average daily SO_2_ concentrations in Hong Kong (mean ± standard deviation: 19.5 ± 13.2) and Chengdu (mean ± standard deviation: 13.8 ± 5.6) and definitions of “cool” in Hong Kong study (November to April) and our study (cut-off points of temperature with the lowest GCV scores). In our study, we found high relative risk (a 10 μg/m^3^ increment associated with 11.22% increase in COPD HAs) and low absolute risk (using WHO air quality guidelines as the reference concentration, 1.14% COPD HAs attributable to SO_2_ exceeding exposure) between SO_2_ and COPD HAs on low temperature days in urban area of Chengdu. This suggested that we can ignore the effect of SO_2_ on COPD HAs because of low absolute risk, or maybe there exists underestimation of absolute risk; in China, assessment of SO_2_ concentration using weaker concentration breakpoints, which will lead to be less severe compared with using the US classification [43]. So, if we had underestimation of SO_2_ concentration, absolute risk on COPD HAs was underestimated because of high relative risk of SO_2_. The influence of SO_2_ on COPD HAs with high relative risk and low absolute risk should be further researched. 

Exposure to 10 μg/m^3^ increment in NO_2_ was positively associated with 5.59% (95% CI: 3.65%, 7.56%) increases in COPD HAs on low temperature days, which was statistically higher than that on moderate days, while this ostensible interact effect was insignificant after adjustment for other pollutant (PM_2.5_, SO_2_ or CO). This phenomenon has also been reported in previous study [44]. Both PM pollutants and NO_2_ were associated with COPD HAs, and they were relatively highly correlated (coefficients > 0.6, Table S2). Therefore, it was usually difficult to examine their individual effects [37].

Regarding the association between CO and COPD HAs, conflicting results were reported. For instance, Cai et al. [45] demonstrated negative associations, with a considerable decrease of 2.97% (95% CI: −4.63%, −1.31%) in COPD HAs per an interquartile range increase (0.6 mg/m^3^) of CO in Shanghai, China. In our analyses, without interaction with temperature, CO per 0.1 mg/m^3^ increment associated with 2.63% (95% CI: 1.79%, 3.49%) increase on COPD HAs. Heterogeneity in the effects estimates of CO may be related with masked effects by combustion of other source-related pollutants in different areas. In Chengdu, CO concentration was mainly an artificial product of incomplete combustion of carbon-containing fuels, such as automobile exhaust and incineration of solid waste. Consistent positive association between CO and COPD HAs in urban areas of Chengdu may be related to possessing the second largest number of vehicles in China and biomass burning in sub-urban areas [46]. 

The elderly population (≥80 years) was more susceptible to COPD HAs, consistent with previous studies, mainly due to pathogen exposure, aging and comorbidity, which has been shown to weaken immune defenses and respiratory function, resulting in respiratory infections [16,40]. Also, this phenomenon might be related with the larger sample size of daily admission counts for the elderly. Males had a little higher but insignificant effect estimate than females in our study, which might be associated with the fact that 66% of men smoke whereas only around 3% of women smoke, but they might be exposed to second-hand tobacco smoke from men smokers and also exposed to household air pollution in China [47]. 

Compared with ratio measures, AF and AN could provide more information on excess burden due to interactions between ambient air pollutants and temperatures [34]. In our study, the highest AF consistently occurred at low temperature levels. When using WHO air quality guidelines as the reference, approximately 7.33% (95% CI: 4.31%, 10.34%) and 6.26% (95% CI: 3.66%, 8.86%) of COPD HAs were attributable to PM_2.5_ and PM_10_ exposures, respectively. Specifically, on low temperature days, 17.30% (95% CI: 12.39%, 22.19%) and 14.72% (95% CI: 10.38%, 19.06%) of COPD HAs were estimated to be attributed to PM_2.5_ and PM_10_, respectively. Similar approaches have been used previously by Chen et al., which estimated that 10.7% of incident influenza cases might have been caused by exposure to ambient PM_2.5_ [12]. Chen et al. assessed 3.34% and 3.96% of emergency hospital visits due to ambient PM_2.5_ and PM_10_ exposures, respectively [48]. Li et al. demonstrated that COPD mortality in Guangzhou may decrease by 4.31% if the level of PM_10_ is reduced to the target values of WHO [34]. 

This study had several strengths. First, instead of seasonal variations [37], we used different cut-off points of daily temperature to select the model with the lowest GCV score [8]. Second, the majority of studies investigating the interaction between ambient air pollution and temperature on health have mainly focused on mortality outcomes. In contrast, HAs is a more sensitive indicator to measure the health response to fluctuations in environmental factors, which covers higher numbers of patients and confers a greater statistical power. Third, we used only primary diagnosis of COPD HAs in urban areas of Chengdu, which could reduce misclassification of outcomes and avoided admission bias [29]. Finally, few studies have evaluated the modification effect of temperature on association between ambient air pollution and COPD-related health disorders in China, especially in basin climate regions, where the air pollutant mixtures and meteorological factors may potentially be quite different due to the unique topography. Chengdu, a typical basin climate city, may greatly benefit from a comprehensive understanding of health effect of interactions between ambient air pollution and temperature [49]. 

Potential limitations should be taken into consideration. First, outdoor fixed monitoring stations to individuals’ exposure resulted in unavoidable exposure measurement error, which is an inherent limitation of epidemiology studies of disease and environment [50]. Second, individual level data on explanatory factors such as smoking, activity patterns and indoor pollutants should be further studied, which may confound the present association. Third, the potential misdiagnosis of COPD should also be considered when interpreting the findings. All tertiary and secondary hospitals enjoy the esteems for quality in all aspects of healthcare, but minor misdiagnosis still exists [51]. In addition, our study was restricted to urban areas of Chengdu, with high population density (>5000 people/km^2^), where air pollution would be higher than that in the resting areas of Chengdu because of automobile exhaust, urbanization, unfavorable diffusion conditions at the bottom of Sichuan basin, secondary pollution, and so on. Also, the study period was only two years because of data unavailability, which limited our research power.

## 5. Conclusions

In conclusion, we found evidence of interactions between ambient air pollutants (PM_2.5_, PM_10_ and SO_2_) and low temperature on COPD HAs in Chengdu. The elderly (≥80 years) and males were more vulnerable to this interaction. The morbidity burden attributable to the interaction between PM and low temperature was particularly high. These findings suggest that it is important to control and reduce the emission of ambient air pollutants in Chengdu, particularly when temperature decreases. Better understanding of these ambient air pollutants and temperature interactions has important implications for planning intervention measures to reduce the risk of hospital admissions for COPD.

## Figures and Tables

**Table 1:** Summary statistics of air pollutants, meteorological variables and COPD hospital admissions in urban areas of Chengdu, China (2015–2016).

	Mean ± SD	Minimum	Percentiles			Maximum
			25	50	75	
Daily COPD HAs (n)	75 ± 32	15	52	70	94	194
COPD HAs by sex (n)						
male	48 ± 21	9	33	45	62	126
female	27 ± 13	3	18	25	33	86
COPD HAs by age (n)						
≥80	29 ± 13	2	19	27	37	88
70–80	25 ± 12	4	17	23	32	69
60–70	15 ± 7	1	9	13	19	43
<60	6 ± 3	1	4	6	8	21
Air pollution levels *						
PM_2.5_ (μg/m^3^ )	57.29 ± 36.75	7.33	30.62	46.48	73.52	232.45
PM_10_ (μg/m^3^ )	94.73 ± 57.07	12.77	53.43	77.50	124.09	339.20
SO_2_ (μg/m^3^ )	13.80 ± 5.61	3.53	9.56	12.75	16.94	34.83
NO_2_ (μg/m^3^ )	50.49 ± 15.21	13.86	39.22	48.05	59.60	105.74
O_3_ (μg/m^3^ )	96.73 ± 55.77	5.60	53.00	86.20	136.60	290.40
CO (mg/m^3^ )	1.07 ± 0.35	0.40	0.82	0.99	1.23	2.69
Meteorological measures						
Temperature (°C)	16.99 ± 7.10	−1.10	10.40	18.00	23.00	30.20
Relative Humidity (%)	80.46 ± 8.87	42.98	74.64	80.80	87.47	98.30

SD: standard deviation; * the daily concentrations of air pollution were calculated as the 24-h mean concentration, except for O_3_, which was calculated as the maximum 8-h moving average.

**Table 2:** Percentage change in COPD hospital admissions per 10 μg/m^3^ increase in air pollutants (CO per 0.1 mg/m^3^ ) by temperature levels in urban areas of Chengdu, China, 2015–2016.

Pollutants ^§^	Temperature ^a^		
	Low	Moderate	High
PM_2.5_	2.90 (2.07, 3.73) *	1.51 (0.70, 2.34) *	1.81 (0.17, 3.46) *
+SO_2_	2.68 (1.83, 3.54) *	1.26 (0.41, 2.11) *	1.56 (−0.09, 3.23)
+NO_2_	2.53 (1.67, 3.39) *	1.11 (0.26, 1.96) *	1.21 (−0.45, 2.89)
+CO	2.25 (1.37, 3.13) *	0.81 (−0.06, 1.69)	0.76 (−0.93, 2.46)
PM_10_	1.73 (1.22, 2.25) *	0.90 (0.42, 1.40) *	1.04 (0.08, 2.00) *
+SO_2_	1.60 (1.06, 2.13) *	0.74 (0.22, 1.26) *	0.89 (−0.08, 1.86)
+CO	1.31 (0.75, 1.86) *	0.46 (−0.08, 1.00)	0.42 (−0.57, 1.43)
SO_2_	17.04 (10.77, 23.66) *	6.54 (1.41, 11.93) *	8.40 (1.46, 15.82) *
+PM_2.5_	11.22 (4.70, 18.14) *	1.79 (−3.58, 7.47)	2.80 (−4.23, 10.36)
+PM_10_	11.54 (5.08, 18.39) *	1.80 (−3.55, 7.46)	2.74 (−4.29, 10.28)
+NO_2_	13.14 (6.81, 19.85) *	2.76 (−2.51, 8.32)	3.87 (−3.07, 11.30)
+CO	11.83 (5.59, 18.44) *	1.77 (−3.41, 7.24)	2.48 (−4.40, 9.85)
NO_2_	5.59 (3.65, 7.56) *	2.87 (1.22, 4.54) *	3.50 (1.42, 5.62) *
+PM_2.5_	3.42 (1.17, 5.72) *	0.94 (−1.00, 2.92)	1.35 (−1.01, 3.76)
+SO_2_	4.82 (2.70, 6.98) *	2.10 (0.24, 4.00) *	2.73 (0.48, 5.02) *
+CO	3.48 (1.37, 5.64) *	0.89 (−0.96, 2.77)	1.19 (−1.08, 3.52)
CO ^#^	2.94 (2.10, 3.79) *	1.84 (1.07, 2.61) *	1.91 (0.93, 2.90) *
+PM_2.5_	2.30 (1.38, 3.22) *	1.25 (0.41, 2.10) *	1.25 (0.20, 2.31) *
+PM_10_	2.34 (1.43, 3.25) *	1.26 (0.43, 2.11) *	1.26 (0.21, 2.31) *
+SO_2_	2.69 (1.81, 3.58) *	1.57 (0.75, 2.40) *	1.64 (0.62, 2.67) *
+NO_2_	2.51 (1.64, 3.39) *	1.38 (0.57, 2.20) *	1.39 (0.37, 2.42) *

^a^ Temperature: °C. Low: daily average temperature ≤ 20th percentile; Moderate temperature: 20th percentile < daily average temperature < 80th percentile; High: daily average temperature ≥ 80th percentile; ^§^ Results are shown on lag 06 for PM_2.5_, lag 05 for PM_10_, lag 05 for SO_2_, lag 05 for NO_2_, and lag 05 for CO; * Statistical significantly (*p* < 0.05); Bolded figures are statistically higher than equivalent estimates in moderate temperature stratum; ^#^ Percentage change in daily COPD hospital admissions per 10 μg/m^3^ increase in air pollutants, except for CO per 0.1 mg/m^3^ .

**Table 3:** Attributable fraction and number (and 95% confidence interval) of COPD hospital admissions due to PM_2.5_, PM_10_ and SO_2_ by temperature levels.

Target Levels (μg/m^3^ ) *	Temperature Level	PM_2.5_		PM_10_		SO_2_	
		AF (%, 95% CI)	AN (No., 95% CI)	AF (%, 95% CI)	AN (No., 95% CI)	AF (%, 95% CI)	AN (No., 95% CI)
WHO (25, 50, 20)	Low	17.30 (12.39, 22.19)	2260 (1619, 2900)	14.72 (10.38, 19.06)	1939 (1368, 2511)	1.14 (0.74, 1.54)	150 (98, 203)
	Moderate	4.77 (2.21, 7.33)	1533 (710, 2356)	4.16 (1.92, 6.41)	1338 (616, 2060)	0.08 (0.02, 0.14)	25 (5, 44)
	High	2.10 (0.21, 4.00)	194 (19, 369)	1.45 (0.12, 2.80)	134 (11, 258)	0 (0, 0)	0 (0, 0)
	Overall	7.33 (4.31, 10.34)	3987 (2348, 5625)	6.26 (3.66, 8.86)	3411 (1995, 4829)	0.32 (0.19, 0.45)	175 (103, 247)
China grade II (75, 150, 150)	Low	5.89 (4.22, 7.57)	770 (552, 989)	3.35 (2.36, 4.33)	441 (311, 570)	0 (0, 0)	0 (0, 0)
	Moderate	0.64 (0.30, 0.99)	206 (96, 317)	0.28 (0.13, 0.44)	91 (42, 140)	0 (0, 0)	0 (0, 0)
	High	0 (0, 0)	0 (0, 0)	0 (0, 0)	0 (0, 0)	0 (0, 0)	0 (0, 0)
	Overall	1.79 (1.19, 2.40)	976 (648, 1306)	0.98 (0.65, 1.30)	532 (353, 710)	0 (0, 0)	0 (0, 0)
50% China grade II (37.5, 75,75)	Low	13.73 (9.84, 17.62)	1794 (1286, 2303)	10.60 (7.48, 13.73)	1397 (986, 1809)	0 (0, 0)	0 (0, 0)
	Moderate	3.08 (1.43, 4.74)	991 (459, 1523)	2.41 (1.11, 3.71)	774 (356, 1192)	0 (0, 0)	0 (0, 0)
	High	0.65 (0.07, 1.24)	60 (6, 114)	0.31 (0.02, 0.60)	29 (2, 55)	0 (0, 0)	0 (0, 0)
	Overall	5.23 (3.22, 7.24)	2845 (1751, 3940)	4.03 (2.46, 5.60)	2200 (1344, 3056)	0 (0, 0)	0 (0, 0)

AF: attributable fraction; CI: confidence interval; AN: attributable number; No.: number of cases; * The target levels of PM_2.5_, PM_10_ and SO_2_ are shown in parentheses.

## Supplementary Materials

The following are available online at http://www.mdpi.com/1660-4601/15/3/492/s1, Table S1: GCV score in different cut-off points of temperature ranging from 5th and 25th in interactive model, Table S2: Spearman’s correlations between the different pollutants and meteorological variables, Figure S1: Percentage changes with 95% confidence interval in daily COPD admissions associated with air pollutants concentrations with different lag days in single-pollutant model, Figure S2: Associations between daily air pollutant concentrations and COPD HAs stratified by varying percentiles of temperature (lag03) cut-off points, Figure S3: Associations between CO/NO_2_ concentrations and daily COPD HAs in low, moderate and high temperature strata by age and gender.
