# Supplementary material for: The Burden of COPD Morbidity Attributable to the Interaction between Ambient Air Pollution and Temperature in Chengdu, China
Source: Int J Environ Res Public Health. 2018 Mar 11;15(3):492. doi: 10.3390/ijerph15030492 (PMC5877037; doi:10.3390/ijerph15030492)
Supplement: Supplementary file 1 [file ijerph-15-00492-s001.pdf]

Supplemental Materials: The Burden of COPD Morbidity Attributable to the Interaction between Ambient Air Pollution and Temperature in Chengdu, China

Hang Qiu, Kun Tan, Feiyu Long, Liya Wang, Haiyan Yu, Ren Deng, Hu Long, Yanlong Zhang and Jingping Pan

Table S1. GCV score in different cut-off points of temperature ranging from 5th and 95th to 25th and 75th percentiles in interactive model.

Model	GCV scores				
	Tem_P5	Tem_P10	Tem_P15	Tem_P20	Tem_P25
$PM_{2.5}$ (lag 06)	3.180382	3.209705	3.129714	3.128002	**3.126682**
PM_{10} (lag 05)	3.194187	3.22229	3.145043	**3.143943**	3.153561
SO_2 (lag 05)	3.27661	3.288076	3.174095	**3.167676**	3.189079
NO_2 (lag 05)	3.262214	3.278444	3.185947	**3.164945**	3.186649
CO (lag 05)	3.200159	3.214341	3.112365	**3.108569**	3.118869

The cut-off points are 5th and 95th for Tem_P5, 10th and 90th for Tem_P10, 15th and 85th for Tem_P15, 20th and 80th for Tem_P20, 25th and 75th percentiles for Tem_P25. Bolded figures are the lowest GCV scores in each pollutant model, when using different cut-off points.

Table S2. Spearman's correlations between the different pollutants and meteorological variables.

	$PM_{2.5}$	SO_2	PM_{10}	CO	NO_2	O_3	Tem	RH
$PM_{2.5}$	1	0.62*	0.97*	0.79*	0.79*	-0.21*	-0.42*	-0.04
SO_2		1	0.66*	0.51*	0.65*	-0.07	-0.11*	-0.16*
PM_{10}			1	0.75*	0.81*	-0.18*	-0.40*	-0.11
CO				1	0.71*	-0.31*	-0.44*	0.07*
NO_2					1	-0.11	-0.23*	-0.08
O_3						1	0.74*	-0.52*
Tem							1	-0.1
RH								1

* $p < 0.05$; Tem: temperature; RH: relative humidity.

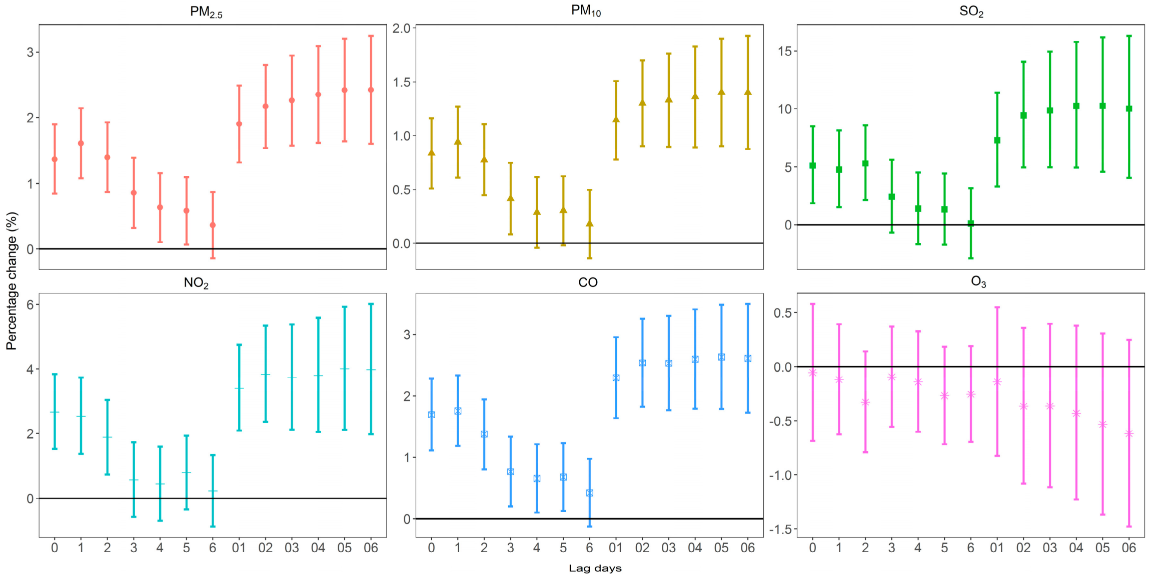

Figure S1. Percentage changes with 95% confidence interval in daily COPD admissions associated with air pollutants concentrations with different lag days in single-pollutant models. The percentage change in daily COPD admissions per 10μg/m³ increase in air pollutants, except for CO per 0.1mg/m³.

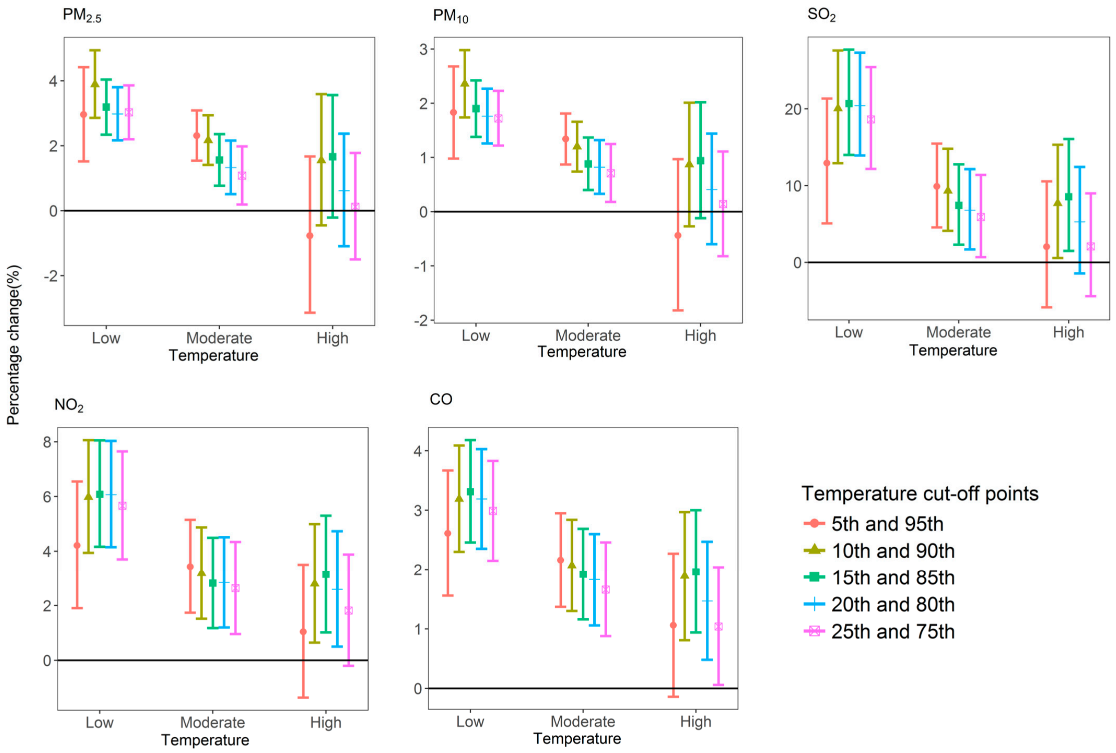

Figure S2. Associations between daily air pollutant concentrations and COPD HAs stratified by varying percentiles of temperature (lag 03) cut-off points.

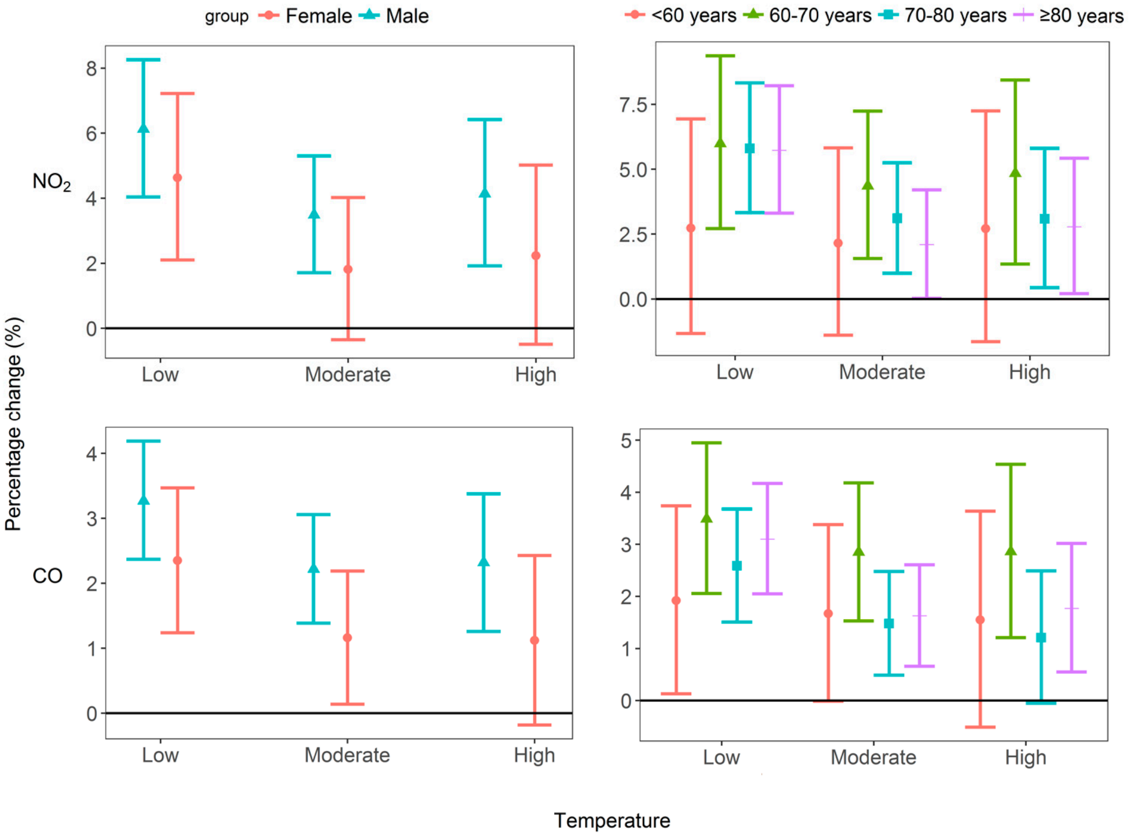

Figure S3. Associations between CO/NO2 concentrations and daily COPD HAs in low, moderate and high temperature strata by age and gender.
